# Elevated NHHR at admission is independently associated with elevated post-stroke depressive symptoms

**DOI:** 10.3389/fpsyt.2026.1724069

**Published:** 2026-06-26

**Authors:** Xiangqi Kong, Xinyue Yuan, Haobo Wang, Mina Zhao, Wei Jing

**Affiliations:** Third Hospital of Shanxi Medical University, Shanxi Bethune Hospital, Shanxi Academy of Medical Sciences, Tongji Shanxi Hospital, Taiyuan, China

**Keywords:** AIS, lipid–inflammation, NHHR, post-stroke depression, risk stratification

## Abstract

**Background:**

Elevated post-stroke depressive symptoms are common among acute ischemic stroke (AIS) survivors and are associated with poor functional recovery. However, readily available biomarkers reflecting lipid–inflammation status remain limited. The ratio of non–high-density lipoprotein cholesterol to high-density lipoprotein cholesterol (NHHR) may integrate atherogenic burden and inflammatory status and thus be associated with depressive symptom burden after stroke.

**Methods:**

We retrospectively analyzed a cohort of 518 Chinese AIS patients. Admission NHHR was calculated from routine lipid panels. Depressive symptoms at 90 days were assessed using the Hamilton Depression Rating Scale (HAMD), and elevated post-stroke depressive symptoms were defined according to the prespecified HAMD threshold. Multivariable logistic regression, restricted cubic spline (RCS) analysis, and subgroup analyses were conducted to examine the association between NHHR and elevated depressive symptoms.

**Results:**

Among the 518 patients, 179 exhibited elevated post-stroke depressive symptoms at 90 days. Higher NHHR levels were independently associated with increased odds of elevated depressive symptoms after adjustment for demographics, stroke severity, cognitive function, inflammatory markers, and coagulation parameters (OR = 1.35, *P* = 0.029). Dose–response trends were observed, and RCS analysis suggested a linear relationship without significant nonlinearity. No significant interactions were found across sex, smoking, alcohol use, hypertension, or diabetes subgroups.

**Conclusions:**

Elevated NHHR at admission was independently associated with elevated post-stroke depressive symptoms at 90 days in Chinese AIS patients. As a simple, cost-effective, and readily obtainable biomarker, NHHR may facilitate early risk stratification and guide individualized interventions.

## 1 Introduction

Post-stroke depressive symptoms are among the most common neuropsychiatric complications in survivors of acute ischemic stroke (AIS), affecting approximately 30% of patients (1). These depressive symptoms not only substantially reduce rehabilitation adherence and quality of life but are also closely linked to poor neurological recovery, higher rates of rehospitalization, and increased mortality (2). Despite their clinical significance, post-stroke depressive symptoms are often under-recognized, and there is an absence of objective, readily obtainable biomarkers for early risk identification, which limits timely intervention (3). Therefore, identifying simple and reproducible predictive indicators to detect high-risk patients at an early stage is of critical importance for improving overall outcomes in AIS survivors.

More recently, the interplay between lipid metabolism disorders and inflammatory responses has been recognized as a key shared mechanism linking cerebrovascular disease and mood disturbances (4, 5). On one hand, dyslipidemia can drive both systemic and neuroinflammation (6, 7); On the other hand, inflammatory processes can alter neurotransmitter metabolism, neuroendocrine function, and neural plasticity (8–10), collectively forming the pathophysiological basis of depressive symptoms following stroke. Therefore, identifying biomarkers that effectively integrate information on lipid metabolism and inflammation is of critical importance.

Non–high-density lipoprotein cholesterol (non-HDL) reflects the overall burden of atherogenic lipoproteins (11), whereas high-density lipoprotein cholesterol (HDL) exerts endothelial-protective effects, antioxidant, and anti-inflammatory (12). The ratio of non-HDL to HDL (NHHR) simultaneously captures both the “atherosclerotic burden” and the “protective lipoprotein” components, enabling a more thorough characterization of lipid–inflammation imbalance. Consequently, NHHR offers a theoretical advantage for risk stratification (13, 14).

Recent large-scale analyses from the U.S. NHANES database have provided key evidence for the clinical relevance of NHHR. First, NHHR is positively linked to greater stroke susceptibility, suggesting its utility for stroke risk assessment (15). Second, NHHR shows a positive correlation with depression in adults, emphasizing the connection between lipid imbalance and mood disorders (16). Third, among stroke survivors, elevated NHHR is significantly associated with the occurrence of PSD, providing population-level evidence for the “lipid imbalance–emotional outcome” pathway (17). Collectively, these findings suggest a potential mechanistic pathway in which elevated NHHR may be linked to increased depressive symptom burden in AIS patients through enhanced pro-inflammatory activity, oxidative stress, and endothelial dysfunction.

However, existing evidence is largely derived from cross-sectional studies in U.S. populations, and cohort data linking hospital-based NHHR measurements to subsequent depressive symptom outcomes are lacking. Accordingly, we hypothesized that elevated NHHR levels during the acute phase are an independent risk factor for depressive symptom burden within 90 days in Chinese AIS individuals. This research explored the relationship between admission NHHR and 90-day depressive symptom burden in a Chinese AIS cohort and to evaluate its potential as an early, simple, and generalizable risk stratification marker.

## 2 Methods

### 2.1 Participants

**Figure 1:**
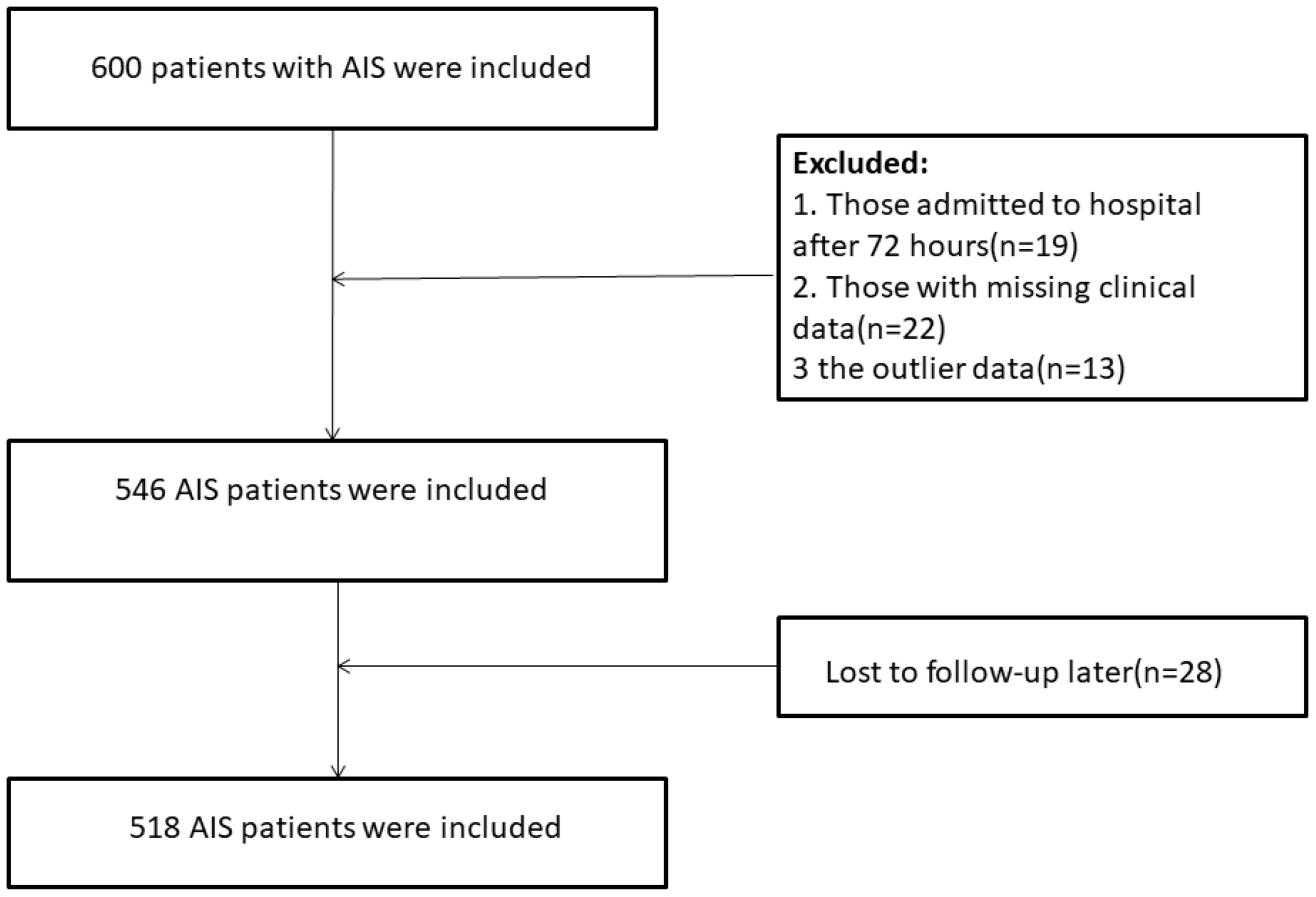
Flowchart of participant selection.

From October 2023 to December 2024, AIS patients admitted to the Department of Neurology, Shanxi Bethune Hospital, were retrospectively analyzed. AIS was defined according to World Health Organization (WHO) criteria, and all diagnoses were confirmed by magnetic resonance imaging (MRI) or brain computed tomography (CT). Patients were included if they were diagnosed with AIS within 72 hours of symptom onset, had a first-ever stroke without prior severe neurological deficits, were older than 18 years, and had complete clinical data with informed consent. Patients were excluded if they had a transient ischemic attack or hemorrhagic cerebrovascular disease, a history of psychiatric disorders, severe aphasia or consciousness impairment preventing reliable assessment, severe cardiopulmonary disease or multi-organ failure, chronic inflammatory disease, incomplete clinical data, or were lost to follow-up. The final analysis comprised 518 eligible patients (**Figure 1**).

### 2.2 Data collection

Baseline demographic and clinical characteristics were recorded, including age, sex, education level, responsible lesion, infarct location, coronary artery disease (CAD), diabetes mellitus (DM), hypertension (HTN), smoking and alcohol use, body mass index (BMI), Montreal Cognitive Assessment (MoCA) score, and National Institutes of Health Stroke Scale (NIHSS) score. Lesion location was determined based on brain MRI findings. The responsible lesion was defined as the acute ischemic lesion identified on MRI that was anatomically consistent with the patient’s presenting neurological deficits. Lesion location was categorized as follows: (1) basal ganglia or lateral ventricle region; (2) brainstem or cerebellum; (3) thalamus; (4) cerebral lobe; and (5) multiple infarctions. Venous blood (2 mL) was collected in EDTA-K2 anticoagulant tubes and analyzed using an automated hematology analyzer (Beckman DXH800) to measure white blood cell (WBC), neutrophil (Neu), monocyte (Mon), lymphocyte (Lym), and platelet (PLT) counts. An additional 5 mL of venous blood was collected in plain tubes, centrifuged, and assayed for uric acid (UA), high-density lipoprotein cholesterol (HDL), total cholesterol (TC), low-density lipoprotein cholesterol (LDL),triglycerides (TG), and homocysteine (Hcy). D-dimer was measured from citrated plasma (1:9 sodium citrate) using the ACL-TOP 750 system (Werfen). The NHHR ratio was calculated as (TC − HDL)/HDL.

### 2.3 Outcome assessment

The primary outcome was elevated post-stroke depressive symptoms at 90 days after admission. Depressive symptoms were assessed during outpatient visits or follow-up using the Hamilton Depression Rating Scale (HAMD) administered by two trained physicians according to standardized clinical procedures. Patients with HAMD scores >7 were classified as having elevated depressive symptoms (18). Stroke severity at admission was evaluated using the NIHSS, with scores <4 indicating mild stroke and scores ≥4 indicating moderate-to-severe stroke (19). Cognitive function was assessed using the MoCA (20).

### 2.4 Statistical analysis

Baseline characteristics were compared according to 90-day elevated depressive symptom status. Normality of continuous variables was evaluated with the Shapiro–Wilk test. Variables following a normal distribution were presented as mean ± standard deviation and analyzed with the Student’s t-test. Non-normally distributed variables were expressed as median (IQR) and compared using the Mann–Whitney U test. Categorical variables were summarized as frequencies (%) and tested using the chi-square method.

Multivariable logistic regression was performed to evaluate the association between NHHR and elevated depressive symptoms. Potential confounders were selected based on clinical relevance, prior literature, and univariable analysis results. Three models were constructed: Model 1, unadjusted; Model 2, adjusted for demographic variables (age and sex); and Model 3, further adjusted for clinically relevant variables, including NIHSS, MoCA, smoking, drinking, education, D-dimer, neutrophil count, and HDL. Multicollinearity diagnostics (**Supplementary Table 2**) indicated that all adjusted covariates had variance inflation factors (VIFs) <5.

Sensitivity analyses were conducted by categorizing NHHR into quartiles and performing trend tests to evaluate robustness. Restricted cubic spline (RCS) models with four knots were used to explore potential nonlinear associations between NHHR and elevated depressive symptoms. Subgroup and interaction analyses were performed by sex, smoking, drinking, hypertension, and diabetes. Each subgroup analysis was adjusted for the same covariates as Model 3, except for the stratification variable itself. All analyses were conducted using R Studio (version 4.4.2) and DecisionLinnc 1.0 software. Two-tailed *P* values below 0.05 were regarded as statistically significant.

## 3 Results

### 3.1 Baseline profiles of the study population

This study included 518 patients, with 339 categorized as without elevated depressive symptoms and 179 as with elevated depressive symptoms (**Table 1**). Comparisons between groups revealed statistically significant differences in sex, smoking, alcohol consumption, NIHSS score, MoCA score, neutrophil count (Neu), D-dimer, HDL, and NHHR (all *P* < 0.05). Specifically, patients in the PSD group were more likely to be male, smokers, and drinkers. They also exhibited higher NIHSS scores, elevated D-dimer levels, and increased NHHR values, while their MoCA scores were significantly lower.

**Table 1:** Baselines characteristics of participants.

Characteristic	Overall N = 518^1^	Without elevated symptoms N = 339^1^	With elevated symptoms N = 179^1^	*p*-value^2^
Sex				<0.001^*^
Female	159 (31%)	128 (38%)	31 (17%)	
Male	359 (69%)	211 (62%)	148 (83%)	
Education				0.960
illiteracy	87 (17%)	58 (17%)	29 (16%)	
Primary schhool	183 (35%)	117 (35%)	66 (37%)	
junior high school	209 (40%)	138 (41%)	71 (40%)	
senior high school and above	39 (7.5%)	26 (7.7%)	13 (7.3%)	
Smoking				0.002
No	216 (42%)	158 (47%)	58 (32%)	
Yes	302 (58%)	181 (53%)	121 (68%)	
Drinking				0.002
No	262 (51%)	188 (55%)	74 (41%)	
Yes	256 (49%)	151 (45%)	105 (59%)	
HTN				0.926
No	204 (39%)	134 (40%)	70 (39%)	
Yes	314 (61%)	205 (60%)	109 (61%)	
DM				0.779
No	360 (69%)	237 (70%)	123 (69%)	
Yes	158 (31%)	102 (30%)	56 (31%)	
CAD				0.465
No	474 (92%)	308 (91%)	166 (93%)	
Yes	44 (8.5%)	31 (9.1%)	13 (7.3%)	
Lesion Location				0.124
Basal ganglia or lateral ventricles	225 (43%)	138 (41%)	87 (49%)	
Brain stem or Cerebellum	147 (28%)	104 (31%)	43 (24%)	
Thalamus	15 (2.9%)	12 (3.5%)	3 (1.7%)	
cerebral lobe	33 (6.4%)	25 (7.4%)	8 (4.5%)	
Multiple infarction	98 (19%)	60 (18%)	38 (21%)	
Stroke Location				0.564
Left	324 (63%)	208 (61%)	116 (65%)	
Right	174 (34%)	116 (34%)	58 (32%)	
Both	20 (3.9%)	15 (4.4%)	5 (2.8%)	
NIHSS	2.50 (1.00, 4.00)	2.00 (1.00, 3.00)	4.00 (3.00, 5.00)	<0.001^*^
Age(years)	63.00 (55.00, 70.00)	63.00 (55.00, 70.00)	63.00 (56.00, 70.00)	0.934
MoCA	25.00 (21.00, 26.00)	25.00 (24.00, 26.00)	21.00 (20.00, 24.00)	<0.001^*^
BMI	22.36 (18.60, 27.09)	22.15 (18.33, 26.78)	22.85 (19.38, 27.88)	0.086
WBC(×10^9/L)	6.35 (5.30, 7.70)	6.40 (5.30, 7.70)	6.30 (5.50, 7.90)	0.605
Lym(×10^9/L)	2.53 (1.97, 3.40)	2.53 (1.95, 3.36)	2.53 (1.97, 3.42)	0.787
Mon(×10^9/L)	0.53 (0.44, 0.64)	0.52 (0.43, 0.64)	0.55 (0.45, 0.65)	0.279
Neu(×10^9/L)	5.91 (4.77, 6.85)	6.02 (5.18, 6.89)	5.59 (3.27, 6.78)	0.003
PLT(×10^9/L)	237.84 (192.86, 283.23)	235.71 (194.28, 283.23)	241.28 (190.57, 283.23)	0.767
D-dimer (ng/mL)	121.50 (76.00, 243.00)	112.00 (69.00, 192.00)	176.00 (92.00, 344.00)	<0.001^*^
TG(mmol/L)	1.47 (1.22, 1.77)	1.47 (1.17, 1.82)	1.47 (1.23, 1.73)	0.834
TC(mmol/L)	4.16 (3.48, 4.86)	4.16 (3.48, 4.92)	4.15 (3.45, 4.76)	0.608
LDL(mmol/L)	2.66 (2.22, 3.16)	2.61 (2.18, 3.21)	2.68 (2.27, 3.10)	0.426
Hcy(umol/L)	16.60 (12.90, 25.90)	16.20 (12.90, 25.60)	17.60 (12.90, 27.10)	0.458
UA(mmol/L)	298.70 (244.40, 385.50)	294.90 (240.30, 378.40)	305.60 (255.30, 388.40)	0.110
HDL(mmol/L)	0.93 (0.81, 1.11)	0.99 (0.88, 1.17)	0.83 (0.75, 0.94)	<0.001^*^
NHHR	3.28 (2.58, 4.15)	3.09 (2.38, 3.92)	3.78 (2.92, 4.58)	<0.001^*^

^1^n (%); Median (Q1, Q3)

^2^Pearson’s Chi-squared test; Wilcoxon rank sum test

HTN, hypertension; DM, diabetes mellitus; CAD, Coronary Artery Disease; WBC, white blood cell; Lym, lymphocyte; Mon, monocyte; Neu, neutrophil; PLT, platelet counts; TG, triglycerides; TC, total cholesterol; LDL, low-density lipoprotein; HDL, high-density lipoprotein; Hcy, homocysteine; UA, uric acid; NHHR, Non-high-density lipoprotein/high-density lipoprotein.

Bonferroni correction was applied for 26 comparisons within this table. The corrected significance threshold is α’ = 0.05/26 ≈ 0.001923. Only variables with p < 0.001923 are considered statistically significant after correction and are marked with *. Nominal p < 0.05 that did not survive correction are reported for transparency but not marked as significant.

Patients with elevated post-stroke depressive symptoms had higher NIHSS scores, higher D-dimer levels, and higher NHHR values, as well as lower MoCA scores compared with those without elevated symptoms.

### 3.2 NHHR and elevated depressive symptoms in AIS patients

Univariable regression analysis (**Supplementary Table 1**) indicated that sex, smoking, alcohol consumption, NIHSS, MoCA, neutrophil count (Neu), D-dimer, HDL, and NHHR were all positively associated with elevated depressive symptoms in AIS patients.

As shown in **Table 2**, in multivariable logistic regression models, NHHR as a continuous variable remained significantly positively associated with elevated depressive symptoms. In the unadjusted Model 1, each one-unit increase in NHHR was associated with higher odds of elevated depressive symptoms (OR = 1.74; 95% CI, 1.48–2.07; *P* < 0.001). After adjusting for age and sex (Model 2), the association remained consistent (OR = 1.74; 95% CI, 1.47–2.07; *P* < 0.001). In the fully adjusted Model 3, which included smoking, alcohol consumption, years of education, NIHSS, MoCA, neutrophil count, D-dimer, and HDL-C, the association remained statistically significant (OR = 1.35; 95% CI, 1.04–1.79; *P* = 0.03).

**Table 2:** Multivariable logistic regression analysis of the association between NHHR and 90-day elevated post-stroke depressive symptoms.

Exposures	Model1 OR (95%CI),*P*-value	Model2 OR (95%CI),*P*-value	Model3 OR (95%CI),*P*-value
NHHR	1.7431 (1.4794, 2.0714),<0.0001	1.7378 (1.4695, 2.0734), <0.0001	1.3528 (1.0350, 1.7851),0.0293
Quartiles			
Q1	Reference	Reference	Reference
Q2	1.4316 (0.8025, 2.5776),0.2265	1.3976 (0.7752, 2.5425),0.2677	1.2312 (0.5233, 2.9317),0.6349
Q3	2.5316 (1.4617, 4.4661),0.0011	2.3613 (1.3481, 4.2085),0.0030	1.5721 (0.6601, 3.8018),0.3094
Q4	4.5246 (2.6359, 7.9522), <0.0001	4.4445 (2.5581, 7.9041), <0.0001	1.7823 (0.7312, 4.4258),0.2068
P for trend	<0.0001	<0.0001	0.1712

Model 1 = no covariates were adjusted.

Model 2 = Model 1 + age,sex, were adjusted.

Model 3 = Model 1 + smoking, drinking, education, NIHSS, MoCA, Neu, D-dimer, HDL were adjusted.

NHHR remained significantly associated with elevated post-stroke depressive symptoms across models, including the fully adjusted model.

When NHHR was categorized into quartiles, a clear dose–response relationship was observed in the unadjusted and demographic-adjusted models. Compared with the lowest quartile (Q1), patients in the highest quartile (Q4) had higher odds of elevated depressive symptoms in Model 1 (OR = 4.52; 95% CI, 2.64–7.95; *P* < 0.001) and Model 2 (OR = 4.44; 95% CI, 2.56–7.90; *P* < 0.001). Trend tests confirmed significant dose-dependent effects (*P* for trend < 0.001). However, in the fully adjusted Model 3, the difference between Q4 and Q1 was no longer statistically significant (OR = 1.78; 95% CI, 0.73–4.43; *P* = 0.21)), and the overall trend was attenuated (*P* for trend = 0.17).

### 3.3 Nonlinear relationship assessment

**Figure 2:**
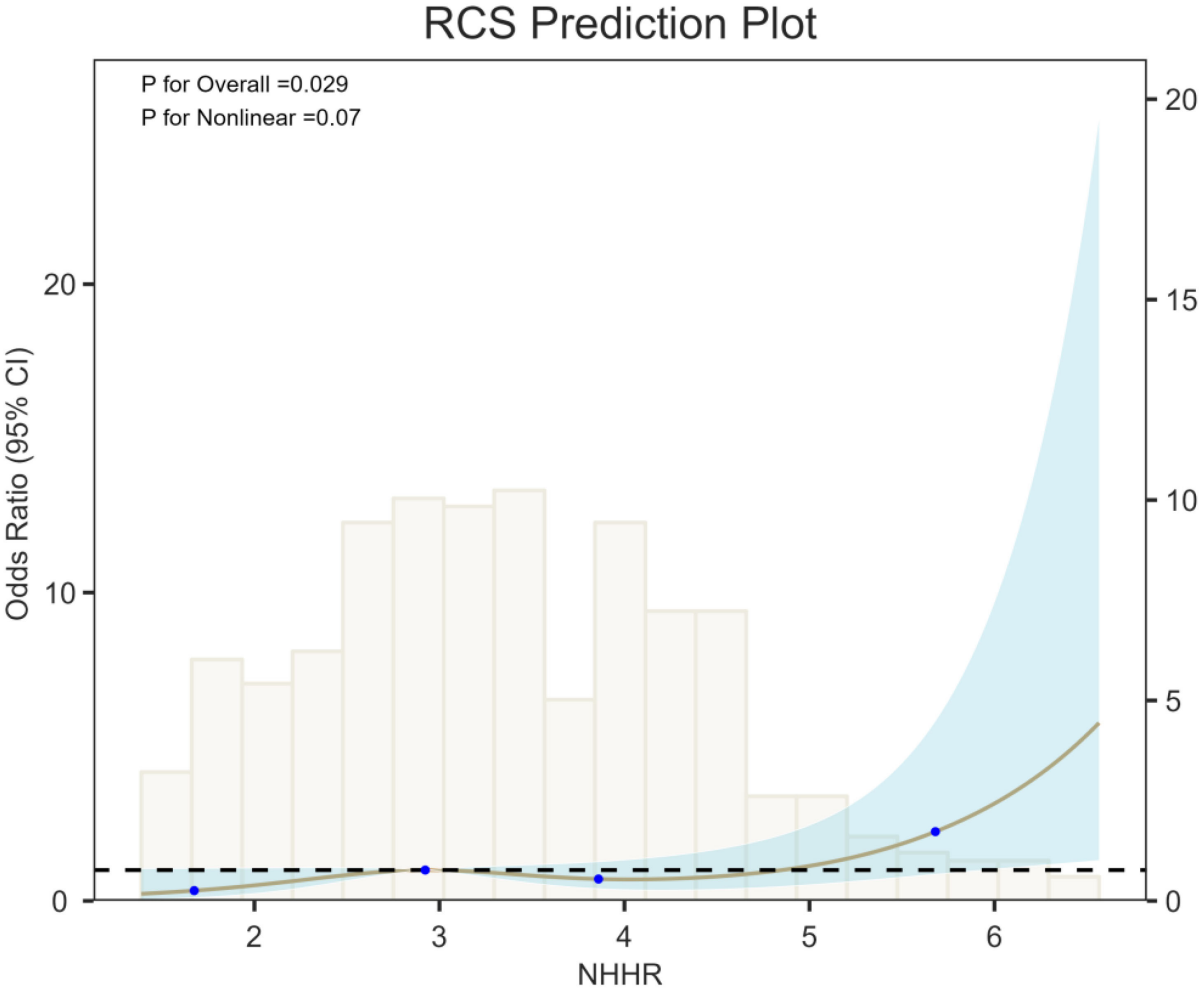
RCS showing the association between NHHR and 90-day elevated post-stroke depressive symptoms. The restricted cubic spline analysis demonstrated a linear positive association between NHHR and 90-day elevated post-stroke depressive symptoms, with no significant nonlinearity detected.

RCS analysis (**Figure 2**) showed a continuous increase in the odds of 90-day elevated depressive symptoms with higher NHHR, demonstrating a significant positive association (*P* for overall trend = 0.029).However, the test for nonlinearity was not significant (*P* = 0.07), indicating no evidence of a nonlinear relationship between NHHR and elevated depressive symptoms at 90 days.

### 3.4 Subgroup analyses

**Figure 3:**
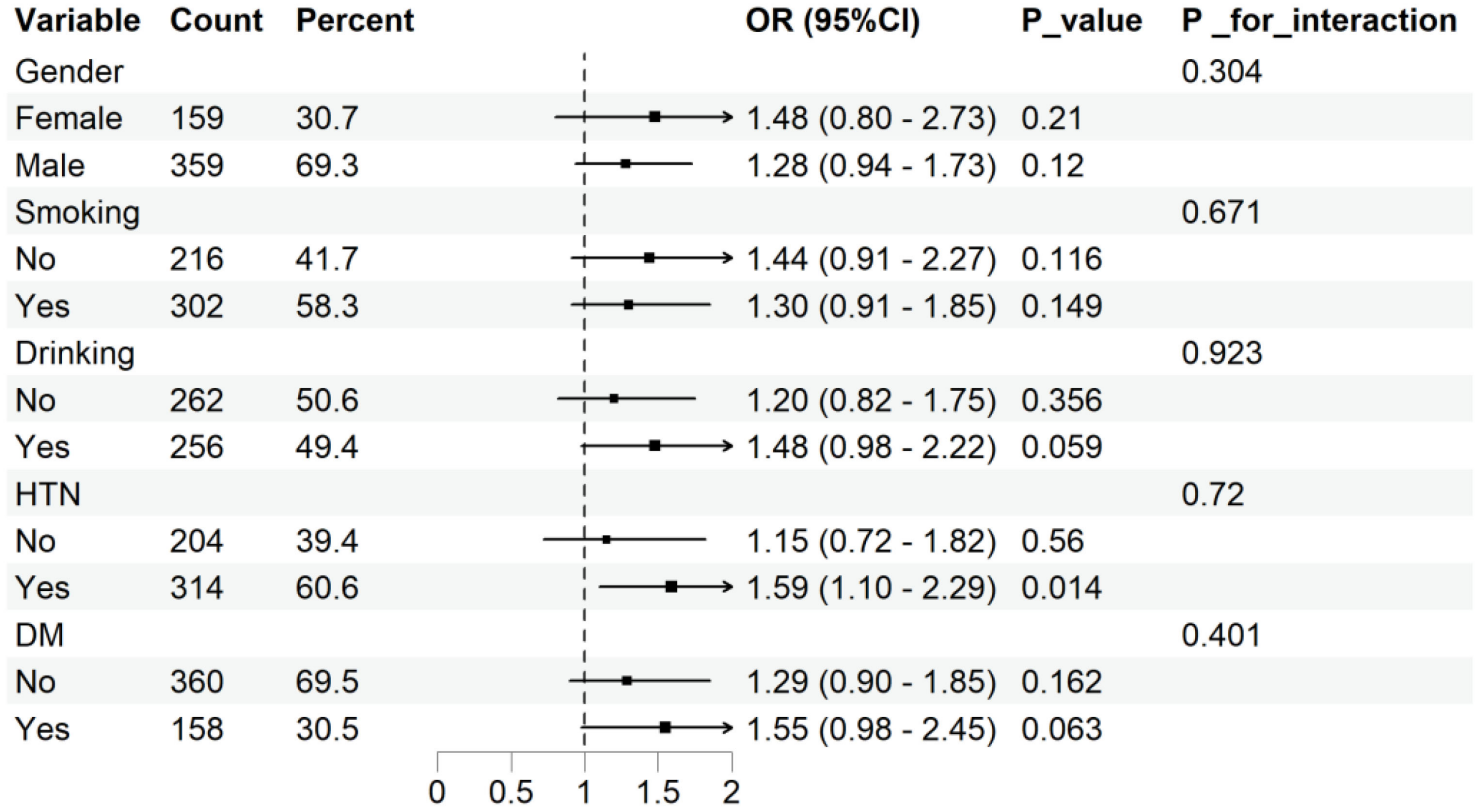
Subgroup analysis for the association between NHHR and 90-day elevated post-stroke depressive symptoms. No significant interactions were observed across predefined subgroups.

Subgroup analyses were conducted according to sex, smoking, drinking, hypertension, and diabetes. No significant effect modification between NHHR and subgroup factors was detected, as depicted in **Figure 3** (*P* for interaction > 0.05).

## 4 Discussion

This investigation represents the first systematic evaluation of the association between admission NHHR and 90-day elevated depressive symptoms among hospitalized Chinese AIS patients. Our results indicate that each one-unit increase in NHHR is associated with approximately a 35% higher odds of elevated depressive symptoms. This association remained significant after comprehensive adjustment for demographic factors, stroke severity, inflammatory markers, and coagulation indices. Restricted cubic spline analysis suggested an approximately linear positive relationship between NHHR and elevated depressive symptoms, with no evidence of nonlinearity. Subgroup analyses revealed no significant interactions. These results indicate that admission NHHR could emerge as an independent risk factor for 90-day elevated depressive symptoms in Chinese AIS patients.

This study provides the first cohort evidence from a Chinese population linking NHHR with elevated depressive symptoms, substantially complementing and extending previous findings. Prior studies investigating the NHHR–PSD relationship have largely relied on cross-sectional analyses of the U.S. NHANES database (17), which have two main limitations. First, the cross-sectional design cannot establish the temporal sequence between elevated NHHR and PSD onset, leaving room for potential reverse causation. Second, lipid metabolism characteristics, such as baseline HDL-C levels, differ significantly between Western and Chinese populations (21, 22), limiting the generalizability of previous results. In contrast, our study employed a retrospective cohort design, using admission NHHR during the acute phase of AIS as the exposure and 90-day elevated depressive symptoms as the outcome. This design clearly establishes the temporal link between exposure and outcome. Focusing on a Chinese cohort, it provides the first quantitative evidence applicable to East Asian populations, offering more targeted guidance for clinical practice.

Second, in terms of association strength and robustness, our findings complement prior research. The NHANES study reported a positive association between NHHR and depressive symptoms among stroke survivors (17), In contrast, after full adjustment for confounders, our study observed a slightly higher OR of 1.3528. This difference may be explained by several factors. First, we strictly included only patients experiencing a first-ever AIS and excluded those with prior neurological deficits, minimizing the influence of chronic brain injury on mood and more accurately capturing the impact of acute-phase lipid imbalance. Second, we adjusted for cognitive function (MoCA), a key confounder, since elevated depressive symptoms frequently co-occurs with cognitive impairment (23); previous studies did not fully control for this factor, potentially underestimating the independent effect of NHHR. Additionally, quartile-based analyses revealed that in unadjusted models, patients in the highest NHHR quartile (Q4) had a 4.5-fold higher odds of elevated depressive symptoms compared with the lowest quartile (Q1) (Model 1: OR = 4.52, *P* < 0.001). Although the difference attenuated after full adjustment (OR = 1.78, *P* = 0.21), the overall trend remained consistent with the primary model. These findings suggest that elevated NHHR represents an important risk signal for depressive symptom burden, with its effect potentially modulated by stroke severity and cognitive status.

While TC showed no group difference, HDL and NHHR were significantly different. This does not necessarily imply that HDL effects are sufficient to cause elevated depressive symptoms. Rather, total cholesterol aggregates both pro- and anti-atherogenic lipoproteins; their opposing influences may obscure a net effect. The significant NHHR difference suggests that the balance between non-HDL and HDL is more relevant. HDL may exert neuroprotective effects, but other lipid fractions and inflammatory pathways likely also contribute. Hence, the absence of a TC difference does not diminish the importance of HDL; instead, it highlights that lipid-associated risk for post-stroke depression is better captured by ratios such as NHHR.

These findings support the concept of a “lipid–inflammation” axis as the plausible link between NHHR and elevated post-stroke depressive symptoms. First, elevated NHHR (high non-HDL combined with low HDL) indicates increased atherogenic lipoprotein burden and impaired protective lipoprotein function. The former promotes macrophage and neutrophil activation, triggering the release of proinflammatory mediators (24), while the latter weakens anti-inflammatory, antioxidant, and blood–brain barrier (BBB) protective effects, thereby exacerbating systemic and central nervous system inflammation (25). These inflammatory signals can cross a compromised BBB, suppress hippocampal neurogenesis, and disrupt monoamine neurotransmitter synthesis (e.g., serotonin and norepinephrine), ultimately leading to depressive symptoms (26). Second, NHHR imbalance induces endothelial dysfunction and impaired cerebral microcirculation, particularly affecting limbic regions that regulate mood. Concurrently, lipid peroxidation and excessive reactive oxygen species contribute to neuronal apoptosis and reduced synaptic plasticity (27). Third, inflammation can stimulate the hypothalamic–pituitary–adrenal (HPA) axis, leading to prolonged cortisol elevation and heightened vulnerability to depression (28). Finally, in the context of acute ischemia–triggered peripheral immune mobilization, elevated NHHR may act as a “secondary hit,” amplifying immune–metabolic cascades and increasing PSD risk (29). Collectively, these mechanisms provide biological plausibility for the observed association between NHHR and post-stroke emotional disturbances.

Interestingly, our findings indicated that male patients were more likely to exhibit elevated post-stroke depressive symptoms, which differs from much of the existing literature reporting a higher prevalence among women. Several factors may account for this discrepancy. First, sociocultural expectations in certain populations may place greater financial and familial responsibility on men, potentially increasing psychological stress following functional impairment. Second, differences in help-seeking behaviors and symptom reporting patterns may vary across cultural contexts. Third, variations in study design, outcome definitions, and sample characteristics may also contribute to inconsistent findings. Therefore, this observation should be interpreted cautiously and warrants further investigation in larger, multicenter studies.

This study has significant translational value. NHHR, derived from routine lipid panels, is inexpensive, easy to measure, and immediately available. Its characteristics align with the principles of precision medicine—simplicity, cost-effectiveness, and scalability—making it a promising screening tool. In particular, NHHR may function as a routinely obtainable admission biomarker to help identify AIS patients who may warrant closer psychological monitoring and supportive interventions, such as counseling and lifestyle modification. The ratio of non-HDL to HDL (NHHR) simultaneously captures both the “atherosclerotic burden” and the “protective lipoprotein” components, enabling a more thorough characterization of lipid–inflammation imbalance. Unlike leukocyte-based inflammatory markers, NHHR is not directly influenced by acute neutrophil elevation caused by stroke-related tissue injury or infectious complications. In addition, because stroke severity itself may affect PSD risk, NIHSS score was further adjusted in the multivariable logistic regression analysis. The persistence of the association after adjustment suggests that NHHR may be independently associated with PSD risk rather than merely reflecting greater stroke severity.

The study has several strengths. First, it provides cohort-based evidence from hospitalized AIS patients in China, addressing a gap in non-Western populations. Second, multiple clinical and laboratory variables were included, allowing systematic adjustment for confounders and yielding robust results. Nevertheless, limitations exist. This is a single-center retrospective study, and causal inferences should be interpreted cautiously. In addition, depressive symptoms were evaluated at a single 90-day time point, which may not fully capture the dynamic longitudinal changes in PSD across different recovery stages after stroke. Because valid HAMD assessment requires survival and adequate communication ability, patients with early death, severe aphasia, or consciousness disturbance were excluded by design. Therefore, our findings apply primarily to patients who are clinically stable enough to complete psychiatric follow-up, and may not generalize to more severe stroke populations. Furthermore, although stroke location was evaluated in the present study, no significant association with post-stroke depressive symptoms was observed in the univariable analysis. However, the relatively broad anatomical classification may not fully reflect the potential effects of specific lesion sites or infarct volume on post-stroke psychiatric manifestations. In addition, information regarding post-stroke anti-inflammatory treatments, antidepressant use during follow-up, rehabilitation intensity, social support, and socioeconomic status was not systematically collected. These factors may influence depressive symptom trajectories and could have introduced residual confounding. Although NIHSS score was adjusted in the multivariable analysis to partially account for stroke severity, residual confounding cannot be completely excluded. In addition, elevated post-stroke depressive symptoms were defined using a HAMD cutoff of ≥8, which reflects symptom burden rather than a formal clinical diagnosis. As a result, some participants may not have met criteria for clinical depression, and the findings should be interpreted accordingly. Future multicenter prospective research is warranted to further validate its prognostic significance.

## 5 Conclusion

In this study of Chinese AIS patients, elevated NHHR at admission emerged as an independent risk factor for 90-day post-stroke depressive symptoms, exhibiting a dose–response relationship. As an easily obtainable clinical parameter, NHHR may serve as a practical adjunctive indicator to help identify patients with increased likelihood of depressive symptom burden and to inform early psychological monitoring and individualized supportive strategies. Future multicenter prospective research is warranted to further validate its prognostic significance and to elucidate the underlying biological mechanisms.

## Data Availability

The original contributions presented in the study are included in the article/**Supplementary Material**. Further inquiries can be directed to the corresponding author.
