# Supplementary material for: Elevated NHHR at admission is independently associated with elevated post-stroke depressive symptoms
Source: Front Psychiatry. 2026 Jun 26;17:1724069. doi: 10.3389/fpsyt.2026.1724069 (PMC13350251; doi:10.3389/fpsyt.2026.1724069)

Catalogue

[Table S1 Univariate logistic regression analysis 2](#_Toc231142631)

[Table S2 Multicollinearity Diagnostics Using Variance Inflation Factor (VIF) 4](#_Toc231142632)

[Figure S1 Dot plot of NHHR in participants without and with elevated symptoms. 5](#_Toc231142633)

## Table S1 Univariate logistic regression analysis

| **Characteristic** | **OR** | **95% CI** | **p-value** |
| --- | --- | --- | --- |
| NIHSS | 2.149 | 1.862, 2.509 | <0.001^*^ |
| Age | 0.9981 | 0.9813, 1.015 | 0.828 |
| MoCA | 0.5858 | 0.5294, 0.6434 | <0.001^*^ |
| BMI | 1.023 | 0.9961, 1.051 | 0.094 |
| WBC | 1.048 | 0.9758, 1.125 | 0.195 |
| Lym | 0.9147 | 0.7649, 1.090 | 0.323 |
| Mon | 1.723 | 0.5652, 5.254 | 0.338 |
| Neu | 0.8340 | 0.7549, 0.9202 | <0.001^*^ |
| PLT | 1.001 | 0.9981, 1.003 | 0.578 |
| D_dimer | 1.001 | 1.000, 1.001 | 0.014 |
| TG | 0.9190 | 0.6402, 1.295 | 0.636 |
| TC | 0.9260 | 0.7716, 1.108 | 0.405 |
| LDL | 1.043 | 0.8256, 1.317 | 0.723 |
| HDL | 0.0298 | 0.0103, 0.0794 | <0.001^*^ |
| UA | 1.001 | 0.9991, 1.003 | 0.339 |
| Hcy | 0.9985 | 0.9871, 1.009 | 0.786 |
| NHHR | 1.743 | 1.479, 2.071 | <0.001^*^ |
| Sex |  |  |  |
| Female | — | — |  |
| Male | 2.896 | 1.876, 4.580 | <0.001^*^ |
| education |  |  |  |
| illiteracy | — | — |  |
| Primary schhool | 1.128 | 0.6619, 1.948 | 0.660 |
| junior high school | 1.029 | 0.6090, 1.762 | 0.916 |
| Senior high school and above | 1.000 | 0.4405, 2.208 | >0.999 |
| Smoking |  |  |  |
| No | — | — |  |
| Yes | 1.821 | 1.251, 2.671 | 0.002 |
| Drinking |  |  |  |
| No | — | — |  |
| Yes | 1.767 | 1.226, 2.555 | 0.002 |
| HTN |  |  |  |
| No | — | — |  |
| Yes | 1.018 | 0.7033, 1.478 | 0.926 |
| DM |  |  |  |
| No | — | — |  |
| Yes | 1.058 | 0.7124, 1.562 | 0.779 |
| CAD |  |  |  |
| No | — | — |  |
| Yes | 0.7781 | 0.3838, 1.496 | 0.466 |
| LesionLocation |  |  |  |
| Basal ganglia or lateral ventricles | — | — |  |
| Brain stem or Cerebellum | 0.6558 | 0.4179, 1.020 | 0.063 |
| Thalamus | 0.3966 | 0.0885, 1.291 | 0.161 |
| cerebral lobe | 0.5076 | 0.2063, 1.131 | 0.114 |
| Multiple infarction | 1.005 | 0.6146, 1.631 | 0.985 |
| StrokeLocation |  |  |  |
| Left | — | — |  |
| Right | 0.8966 | 0.6059, 1.319 | 0.582 |
| Both | 0.5977 | 0.1905, 1.587 | 0.331 |

Note:Univariate logistic regression was performed separately for each independent variable. For categorical variables, odds ratios (OR) and p-values are shown for each category relative to the reference group. Bonferroni correction was applied for 31 independent tests (each row in the table represents one test). The corrected significance threshold is α' = 0.05/32 ≈ **0.0015625**. Only rows with p < 0.001613 are considered statistically significant after correction and are marked with **. Nominally significant results (p < 0.05) that did not survive correction (e.g., D‑dimer, Smoking, Drinking) are reported without significance markers.*

## Table S2 Multicollinearity Diagnostics Using Variance Inflation Factor (VIF)

| Variable Name | VIF |
| --- | --- |
| NIHSS | 1.16 |
| Age | 1.80 |
| Sex | 1.59 |
| education | 2.08 |
| Smoking | 2.04 |
| Drinking | 1.62 |
| MoCA | 1.29 |
| Neu | 1.07 |
| D-dimer | 1.05 |
| NHHR | 1.36 |
| HDL | 1.40 |

## Figure S1 Dot plot of NHHR in participants without and with elevated symptoms.


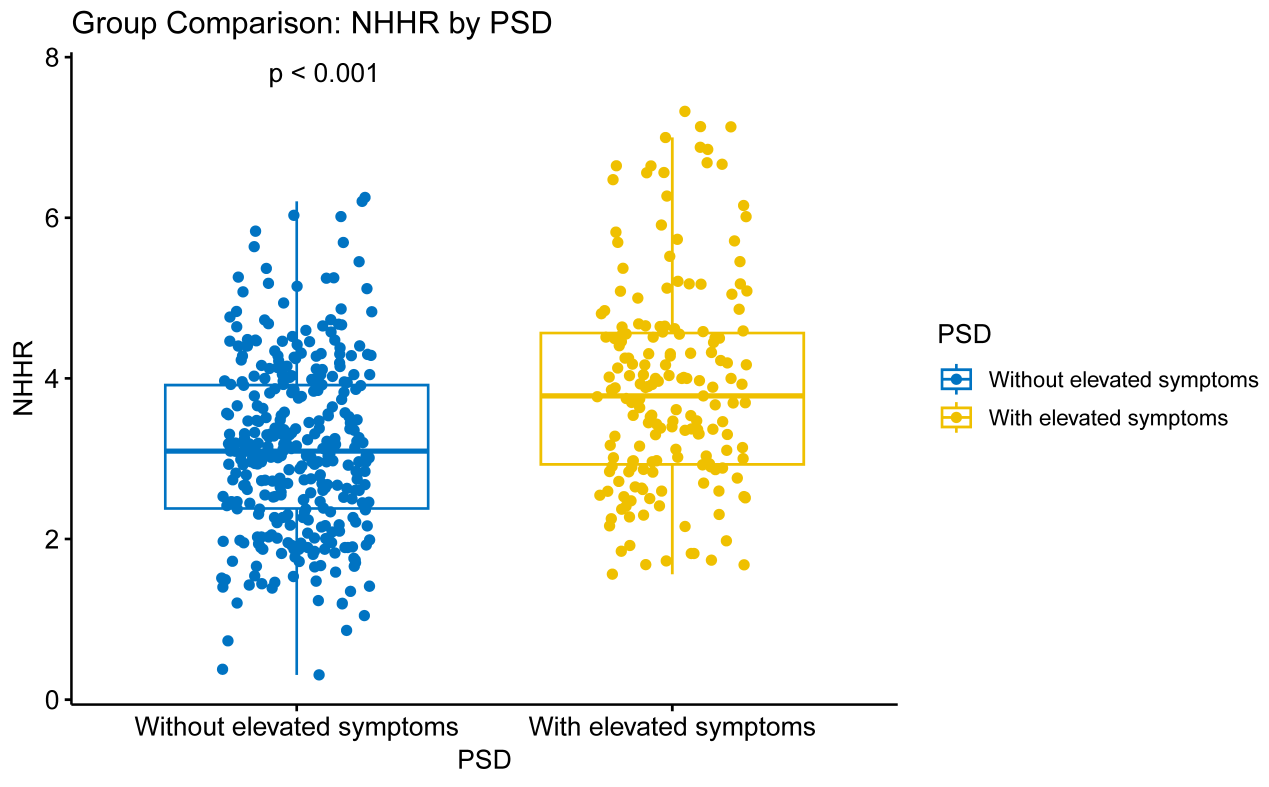

Supplement: Supplementary file 1 [file Supplementaryfile1.docx]
